# Pattern of disease progression during third-line or later chemotherapy with nivolumab associated with poor prognosis in advanced gastric cancer: a multicenter retrospective study in Japan

**DOI:** 10.1007/s10120-022-01349-y

**Published:** 2022-11-01

**Authors:** Masahiko Aoki, Shigenori Kadowaki, Naoki Takahashi, Takeshi Suzuki, Kotoe Oshima, Takayuki Ando, Yoshiyuki Yamamoto, Kentaro Kawakami, Yosuke Kito, Toshihiko Matsumoto, Keitaro Shimozaki, Yasuhiro Miyazaki, Toshifumi Yamaguchi, Michitaka Nagase, Takao Tamura, Yusuke Amanuma, Taito Esaki, Yuji Miura, Kohei Akiyoshi, Eishi Baba, Akitaka Makiyama, Yuji Negoro, Koji Nakashima, Naotoshi Sugimoto, Kengo Nagashima, Hirokazu Shoji, Narikazu Boku

**Affiliations:** 1 Department of Gastrointestinal Medical Oncology, National Cancer Center Hospital, Tokyo, Japan; 2 Department of Early Clinical Development, Graduate School of Medicine, Kyoto University, Kyoto, Japan; 3 Department of Clinical Oncology, Aichi Cancer Center Hospital, Aichi, Japan; 4 Division of Gastroenterology, Saitama Cancer Center, Saitama, Japan; 5 Department of Gastroenterology, The Cancer Institute Hospital, Tokyo, Japan; 6 Department of Gastrointestinal Oncology, Shizuoka Cancer Center, Shizuoka, Japan; 7 Third Department of Internal Medicine, University of Toyama, Toyama, Japan; 8 Department of Gastroenterology, Faculty of Medicine, University of Tsukuba, Ibaraki, Japan; 9 Department of Medical Oncology, Keiyukai Sapporo Hospital, Hokkaido, Japan; 10 Department of Medical Oncology, Ishikawa Prefectural Central Hospital, Ishikawa, Japan; 11 Department of Internalmedicine, Himeji Red Cross Hospital, Hyogo, Japan; 12 Cancer Treatment Center, Kansai Medical University Hospital, Osaka, Japan; 13 Division of Gastroenterology and Hepatology, Depart of Internal Medicine, Keio University School of Medicine, Tokyo, Japan; 14 Department of Surgery, Osaka General Medical Center, Osaka, Japan; 15 Cancer Chemotherapy Center, Osaka Medical and Pharmaceutical University, Osaka, Japan; 16 Department of Medical Oncology, Saku Central Hospital Advanced Care Center, Nagano, Japan; 17 Department of Medical Oncology, Kindai University Nara Hospital, Nara, Japan; 18 Clinical Trial Promotion Department, Chiba Cancer Center, Chiba, Japan; 19 Department of Gastrointestinal and Medical Oncology, National Hospital Organization Kyushu Cancer Center, Fukuoka, Japan; 20 Department of Medical Oncology, Toranomon Hospital, Tokyo, Japan; 21 Department of Medical Oncology, Osaka City General Hospital, Osaka, Japan; 22 Department of Oncology and Social Medicine, Kyushu University Graduate School of Medical Sciences, Fukuoka, Japan; 23 Department of Hematology/Oncology, Japan Community Healthcare Organization Kyushu Hospital, Fukuoka, Japan; 24 Cancer Center, Gifu University Hospital, Gifu, Japan; 25 Division of Gastroenterological Medicine, Kochi Health Sciences Center, Kochi, Japan; 26 Department of Clinical Oncology, University of Miyazaki Hospital, Miyazaki, Japan; 27 Department of Genetic Oncology, Osaka International Cancer Institute, Osaka, Japan; 28 Biostatistics Unit, Clinical and Translational Research Center, Keio University Hospital, Tokyo, Japan; 29 Department of Oncology and General Medicine, IMSUT Hospital, Institute of Medical Science, University of Tokyo, 4-6-1 Shiroganedai, Minato-Ku, Tokyo, 108-8639 Japan

**Keywords:** Hyperprogressive disease, Gastric cancer, Nivolumab

## Abstract

**Background:**

Accelerated tumor growth during immunotherapy in pre-existing measurable lesions, hyperprogressive disease (HPD), has been reported. However, progression of non-measurable lesions and new lesions are frequently observed in patients with advanced gastric cancer (AGC).

**Methods:**

This retrospective study involved AGC patients at 24 Japanese institutions who had measurable lesions and received nivolumab after ≥ 2 lines of chemotherapy. HPD was defined as a ≥ two-fold increase in the tumor growth rate of measurable lesions. The pattern of disease progression was classified according to new lesions in different organs and ascites appeared/increase of ascites.

**Results:**

Of 245 patients, 147 (60.0%) showed progressive disease (PD) as the best response and 41 (16.7%) showed HPD during nivolumab monotherapy. There was no significant difference in overall survival (OS) between patients with HPD and those with PD other than HPD (median OS 5.0 vs 4.8 months; hazard ratio [HR] 1.0, 95% confidence interval [CI] 0.6–1.5; *p* = 1.0). Fifty-three patients developed new lesions in different organs and 58 had appearance/increase of ascites; these patients showed shorter OS than those without each of these features (median OS 3.3 vs 7.1 months, HR 1.8, 95% CI 1.2–2.7, *p* = 0.0031 for new lesions, and 3.0 vs 7.8 months, HR 2.6, 95% CI 1.8–3.8, *p* < 0.0001 for ascites). Thirty-one patients who had both features showed the worst prognosis (median OS 2.6 months).

**Conclusions:**

New lesions in different organs and appearance/increase of ascites, rather than the original definition of HPD, are the patterns of disease progression associated with poor prognosis in AGC patients receiving nivolumab whose best response was PD.

**Supplementary Information:**

The online version contains supplementary material available at 10.1007/s10120-022-01349-y.

## Introduction

Gastric cancer is the fifth most common malignancy and the fourth leading cause of cancer-related death worldwide. The incidence of gastric cancer is two-fold higher in men than in women. In several Asian countries, gastric cancer is the most common cancer in men and the leading cause of cancer-related death [1]. Japan and Korea have a nationwide screening system for gastric cancer. However, with the exception of these two countries, many patients with gastric cancer have unresectable disease at the time of diagnosis, and the cure rate remains low even after surgical resection with or without perioperative adjuvant chemotherapy. The clinical outcome of advanced gastric cancer (AGC) that is unresectable or recurrent remains poor, with median overall survival (OS) of around 1 year despite the various chemotherapeutic agents available.

Several guidelines recommend a combination of platinum and fluoropyrimidine as the first-line treatment for AGC [2–6] with addition of trastuzumab in patients who are positive for human epidermal growth factor receptor 2 [7]. The recommended second-line treatment is a combination of paclitaxel and ramucirumab, a vascular endothelial growth factor receptor 2 antibody [8]. Depending on the patient’s general condition, the recommendations for third-line treatment include trifluridine/tipiracil (TFTD) [9], trastuzumab deruxtecan (T-DXd) [10], irinotecan[11–14], and an immune checkpoint inhibitor (ICI) targeting the programmed cell death (PD)-1 protein, such as nivolumab [15] or pembrolizumab [16–18]. Recently, considerable progress has been made. In the CheckMate-649 study, nivolumab plus chemotherapy achieved significant improvements in OS and progression-free survival (PFS) not only in patients with a combined positive score of PD-ligand 1 ≥ 5 but also in all randomized patients [19]. Accordingly, nivolumab has been approved for use in combination with chemotherapy as first-line treatment for AGC.

However, ICIs may cause a rapid type of tumor growth known as hyperprogressive disease (HPD) [20]. HPD was originally defined as a ≥ two-fold increase in the tumor growth rate (TGR), assessed as the change in volume of a pre-existing measurable lesion per unit time [21], compared with that at the evaluation of disease progression during the previous line of treatment. HPD has attracted attention because it is reported to be associated with poor prognosis. Although the definition of HPD varies slightly from study to study, there have been several reports of HPD during anti-PD-1/PD-L1 therapy. In one study, 12 of 131 patients (9%) with various types of cancer, including melanoma and lung, renal, and colorectal cancer, developed HPD [20], as did 10 of 34 patients (29%) with head and neck cancer [22] and 56 of 406 patients (14%) with lung cancer [23]. There have also been reports on AGC in which 13 of 62 patients (21%) [24], 10 of 34 patients (29%) [25] and 45 of 219 patients (20.5%) [26] developed HPD that was associated with poor prognosis.

Approximately 30%–50% of patients with AGC present with peritoneal metastasis, which is a typical pattern of disease progression in patients with AGC and is well known to be associated with poor prognosis. However, peritoneal metastasis is not included in the original definition of HPD because it is not a measurable lesion. Moreover, there has also been a report suggesting that disease progression accompanied by new lesions has a strong negative impact on the prognosis in patients with AGC [27]. Given the lack of difference in the biological mechanism of ICI-induced rapid progression between measurable lesions and non-measurable/new lesions, exclusion of peritoneal metastasis and new lesions from the definition of HPD may lead to underestimation of accelerated tumor growth during treatment with ICI in patients with AGC.

The aims of this study were to (1) determine the prevalence, background characteristics, and clinical outcomes of HPD in patients with AGC receiving nivolumab monotherapy as salvage treatment and (2) investigate the pattern of disease progression associated with poor prognosis, focusing on peritoneal metastasis and new lesions.

## Methods

### Patients and treatment

This multicenter retrospective study involved patients with AGC who received nivolumab 3 mg/kg or 240 mg/body intravenously every 2 weeks as a third-line or later treatment at 24 participating Japanese institutions between September 2017 and October 2018.

The inclusion criteria were as follows: age ≥ 20 years; histologically confirmed advanced unresectable or recurrent gastric/gastro-esophageal junction adenocarcinoma; refractory to or intolerant of at least 2 previous lines of chemotherapy (must be refractory to the immediately preceding chemotherapy); at least 1 measurable lesion according to Response Evaluation Criteria in Solid Tumors (RECIST) version 1.1; and radiologic images available at 2 time points for evaluating disease progression during previous chemotherapy, the latter of which was used as the baseline for evaluation of the response to nivolumab in some patients, and those for first evaluation of response after initiating nivolumab. Patients who had received previous immunotherapy were excluded. The study was approved by the ethics committee of the National Cancer Center and by all participating institutions.

### Tumor growth rate

Tumor response was assessed according to RECIST version 1.1. The TGR was assessed by comparing the two images obtained during the immediately preceding therapy and during treatment with nivolumab, respectively.

TGR was calculated using the following method. Taking *D* as the sum of the largest diameters of the target lesions as per RECIST version 1.1 (new lesions and non-measurable lesions were not included) and virtually representing a single lesion with size *D*, the tumor volume (*V*) is approximated as *V* = 4/3 × *π* × *R*^3^, where *R* is the radius (*D*/2) of the sphere. Assuming the tumor grows at an exponential rate, the tumor volume at time *t* (Vt) can be calculated as *Vt* = *V*0 exp(TG·*t*), where *V*0 is the volume at baseline and t is the interval time (months) between successive CT scans. Tumor growth (TG) is then calculated as TG = 3 Log(*Dt*/*D*0)/*t*. Finally, TGR, which is the percentage increase in tumor volume per month, is obtained using the following formula: TGR = 100 [exp(TG) − 1] [20, 28–30]. The TGR during the previous therapy and that during treatment with nivolumab were compared, and an increase in TGR of ≥ two-fold was defined as HPD [20].

### Non-measurable lesions and appearance of new lesions

When evaluating disease progression during treatment with nivolumab, we also investigated factors not included in the original definition of HPD, namely, new lesions appearing in different organs other than those involved before initiating nivolumab and ascites (representing peritoneal metastasis). Definition of appearance/increase of ascites was based on the Response Evaluation Criteria in Solid Tumors (RECIST), which was judged as unequivocal disease progression by each physician. Patterns of progression were classified as follows according to whether new lesions developed in different organs or not and whether ascites appeared/increased or not: group (G) 1 (−/−), G 2 (+/−), G 3 (−/+), and G 4 (+/+).

### Statistical analysis

Categorical valuables were compared using Fisher’s exact test and continuous variables using the *t* test. The neutrophil-to-lymphocyte ratio (NLR) was calculated from the laboratory data obtained immediately before initiating nivolumab. Receiver-operating characteristic curves was used to determine the optimum cut-off value for NLR in association with HPD status. The cut-off for tumor size was set at the median value. Upper limit of normal was cut-off value for alkaline phosphatase (ALP), which was included in the Japan Clinical Oncology Group (JCOG) prognostic factor [31]. OS was defined as the time from initiation of nivolumab until the date of death from any cause or censored at the latest follow-up for surviving patients. PFS was defined as the time from initiation of nivolumab until detection of disease progression or death, and survivors without disease progression were censored at the last contact. Survival functions of OS and PFS were estimated using the Kaplan–Meier method and compared using the log-rank test. Hazard ratios (HRs) were estimated using a univariable Cox proportional hazards model. Adjusted odds ratios (OR) for inability to receive subsequent chemotherapy after disease progression were obtained by multivariable logistic regression analysis. All statistical analyses were performed using EZR software for Windows (version 1.37) and SAS version 9.4 (SAS Institute Incorporated, Cary, NC, USA). A *p* value of < 0.05 was considered statistically significant.

## Results

### Patient characteristics

The study population consisted of 245 patients at 24 hospitals who received nivolumab between September 2017 and October 2018 and for whom the TGR immediately before and during nivolumab therapy could be calculated. Baseline characteristics at initiation of nivolumab are shown in Table 1. Median age was 69 years (range 29–94) and 186 patients (75.9%) were male. Eastern Cooperative Oncology Group performance status was 1 or 2 in 176 patients (71.9%). Ninety-three patients (38.0%) had been treated with 3 or more lines of chemotherapy. Peritoneal metastasis was observed in 107 patients (43.7%).

**Table 1:** Patient characteristics at initiation of nivolumab

Total, *n* = 245			
Sex		Age, years	
Male	186 (75.9%)	Median (range)	69 (29–94)
Female	59 (24.1%)		
Performance status		Histological type	
0	65 (26.5%)	Intestinal	133 (54.3%)
≥ 1	176 (71.9%)	Diffuse	100 (40.8%)
Unknown	4 (1.6%)	Unknown	12 (4.9%)
HER2 status		Disease status	
Negative	167 (68.2%)	Recurrent	100 (40.8%)
Positive	66 (26.9%)	Stage IV	145 (59.2%)
Unknown	12 (4.9%)		
Peritoneal metastasis		Liver metastasis	
No	138 (56.3%)	No	115 (47.7%)
Yes	107 (43.7%)	Yes	130 (53.1%)
Metastatic sites, *n*		Previous lines of chemotherapy, *n*	
1	70 (28.6%)	< 3	152 (62.0%)
≥ 2	175 (71.4%)	≥ 3	93 (38.0%)
Prior ramucirumab treatment		NLR	
No	52 (21.2%)	< 1.8	82 (33.5%)
Yes	193 (78.8%)	≥ 1.8	161 (65.7%)
		Unknown	2 (0.8%)

*HER2* human epidermal growth factor receptor 2, *NLR* neutrophil-to-lymphocyte ratio

### Effect of HPD on clinical outcomes in AGC

**Fig. 1:**
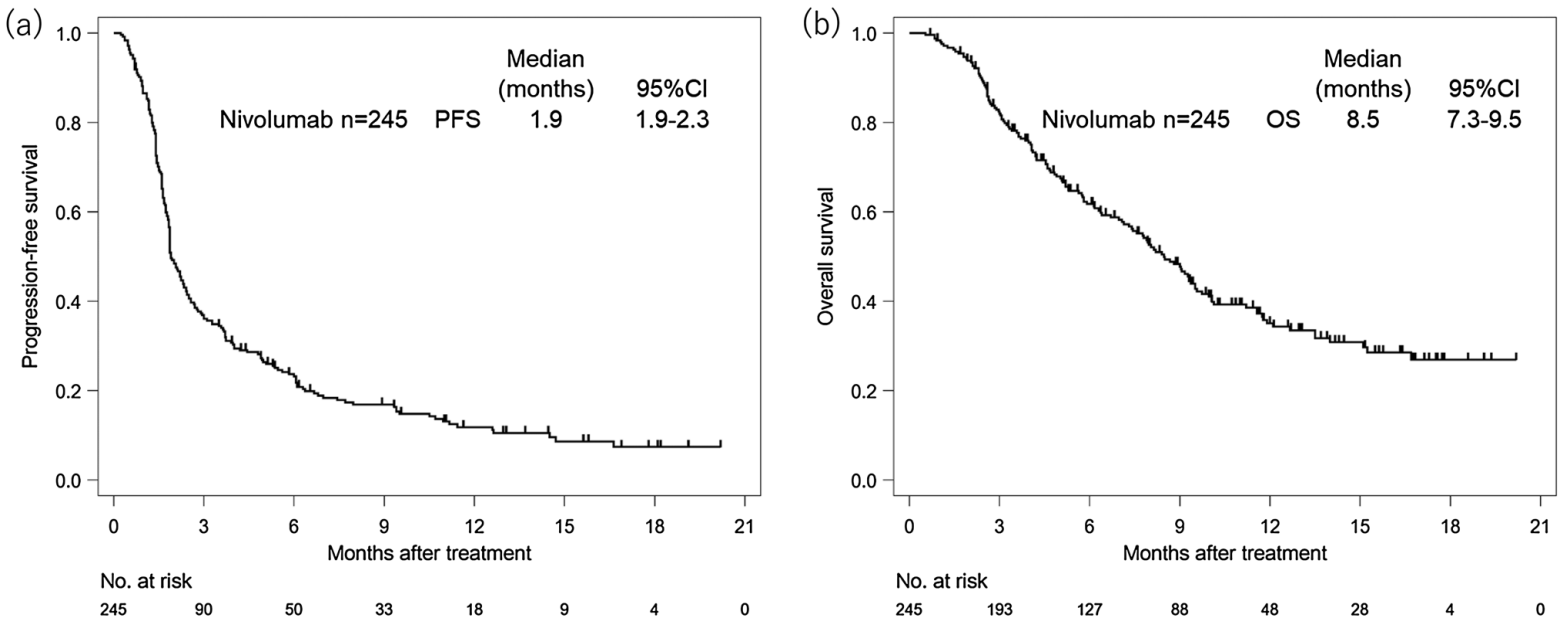
Kaplan–Meier plots showing progression-free survival (PFS) and overall survival (OS). **a** PFS curves after initiation of nivolumab. **b** OS curves after initiation of nivolumab

After initiation of nivolumab, the median follow-up duration in survivors was 13.0 months (range 11.6–14.2). Median OS was 8.5 months (95% confidence interval [CI] 7.3–9.5) and median PFS was 1.9 months (95% CI 1.9–2.3) (Fig. 1). Of the 245 patients, 36 achieved a partial response (PR), including 2 patients showing pseudo progression (1.0%) at the first evaluation and tumor shrinkage thereafter. No patient achieved a complete response, giving a response rate of 14.7% (95% CI 10.5–19.8). One hundred forty-seven patients (60.0%) showed PD as their best response, in whom median PFS was 1.5 months (95% CI 1.4–1.6) and OS was 4.8 months (95% CI 4.1–6.4). In these 147 patients with PD as the best response, 41 were classified as having HPD (Supplementary Table 1). There were no significant differences in baseline characteristics at initiation of nivolumab between the 41 patients with HPD and the 106 patients with PD other than HPD (non-HPD) (Table 2). Forty-seven (44.3%) of the 106 patients with non-HPD and 19 (46.3%) of the 41 patients with HPD received subsequent chemotherapy after disease progression (Supplementary Table 2).

**Table 2:** Patient characteristics at initiation of nivolumab according to HPD and PD (non-HPD) status

Total, *n* = 147	HPD	PD (non-HPD)	*p* value
	*n* = 41	*n* = 106	
Sex			
Male	33 (80.5%)	75 (70.8%)	0.299
Female	8 (19.5%)	31 (29.2%)	
Age, years			
Median (range)	66 (29–83)	69 (41–94)	0.0577
Performance status			
0	11 (26.8%)	26 (24.5%)	0.832
≥ 1	29 (70.7%)	79 (74.5%)	
Unknown	1 (2.5%)	1 (1.0%)	
Histological type			
Intestinal	17 (41.5%)	61 (57.5%)	0.0923
Diffuse	23 (56.1%)	42 (39.6%)	
Unknown	1 (2.4%)	3 (2.9%)	
HER2 status			
Negative	26 (63.4%)	78 (73.6%)	0.405
Positive	13 (31.7%)	27 (25.5%)	
Unknown	2 (4.9%)	1 (0.9%)	
Disease status			
Recurrent	15 (36.6%)	40 (37.7%)	1
Stage IV	26 (63.4%)	66 (62.3%)	
Peritoneal metastasis			
No	24 (58.5%)	55 (51.9%)	0.58
Yes	17 (41.5%)	51 (48.1%)	
Liver metastasis			
No	13 (31.7%)	38 (35.8%)	0.702
Yes	28 (68.3%)	68 (64.2%)	
Metastatic sites, *n*			
1	11 (26.8%)	23 (21.7%)	0.519
≥ 2	30 (73.2%)	83 (78.3%)	
Previous lines of chemotherapy, *n*			
< 3	25 (61.0%)	64 (59.0%)	1
≥ 3	16 (39.0%)	42 (41.0%)	
Prior ramucirumab treatment			
No	9 (22.0%)	24 (22.6%)	1
Yes	32 (78.0%)	82 (77.4%)	
Tumor size, mm			
< 41.3	16 (39.0%)	49 (46.2%)	0.464
≥ 41.3	25 (61.0%)	57 (53.8%)	
Alkaline phosphatase, U/L			
< 350	20 (48.8%)	62 (58.5%)	0.458
≥ 350	21 (51.2%)	43 (40.6%)	
Unknown		1 (0.9%)	
NLR			
< 1.8	18 (43.9%)	33 (31.1%)	0.125
≥ 1.8	22 (53.7%)	73 (68.9%)	
Unknown	1 (2.4%)		

*HER2* human epidermal growth factor receptor 2, *HPD* hyperprogressive disease, *NLR* neutrophil-to-lymphocyte ratio, *PD* progressive disease

**Fig. 2:**
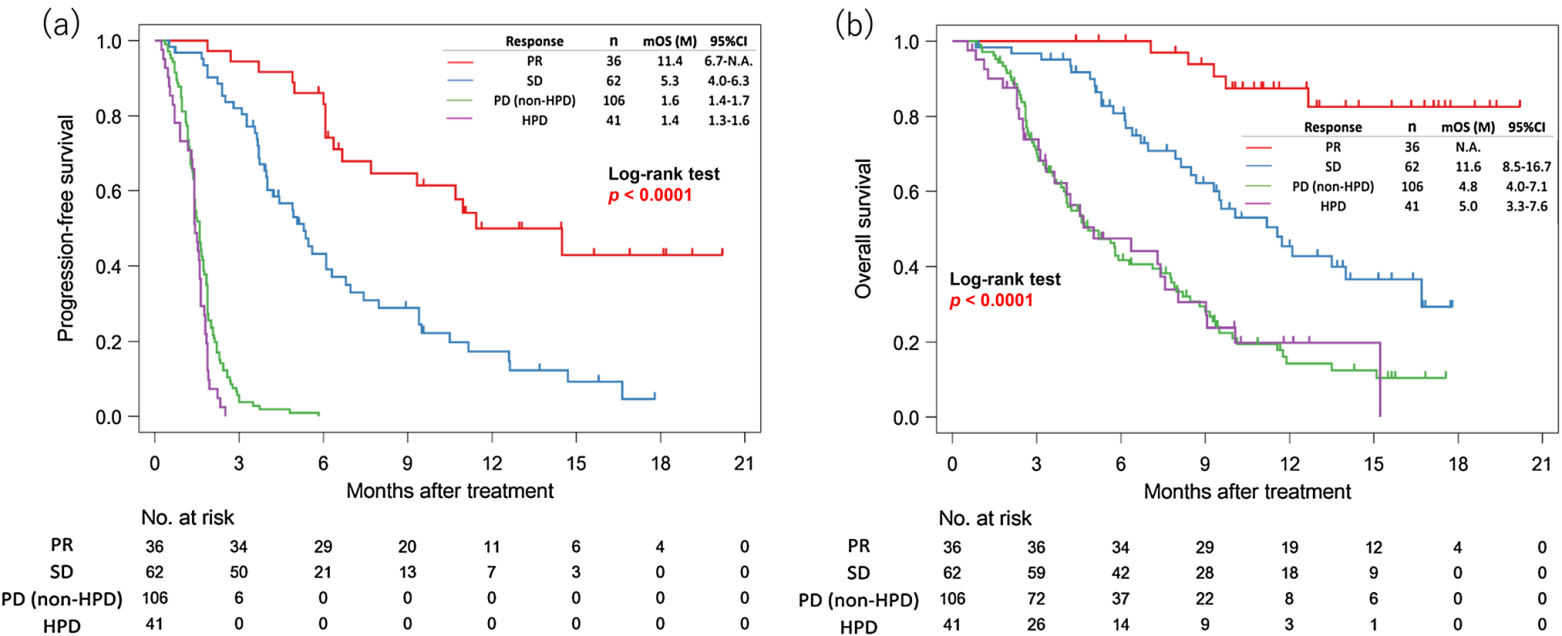
Kaplan–Meier plots showing progression-free survival (PFS) and overall survival (OS). Purple lines indicate patients with hyperprogressive disease (HPD), green lines indicate patients with progressive disease (PD) (non-HPD), blue lines indicate patients with stable disease (SD), and red lines indicate patients with a partial response (PR). **a** PFS curves after initiation of nivolumab. **b** OS curves after initiation of nivolumab

Next, we compared the prognosis of patients with HPD with that of patients with non-HPD, stable disease (SD), or PR (Fig. 2). Median PFS was 1.4 months (95% CI 1.3–1.6) in patients with HPD, 1.6 months (95% CI 1.4–1.7) in those with non-HPD, 5.3 months (95% CI 4.0–6.3) in those with SD, and 11.4 months (95% CI 6.7–NA) in those with PR. Median OS was 5.0 months (95% CI 3.3–7.6) in patients with HPD, 4.8 months (95% CI 4.0–7.1) in those with non-HPD, 11.6 months (95% CI 8.5–16.7) in those with SD, and not reached in those with PR. Although the median PFS was slightly shorter in the 41 patients with HPD than in the 106 with non-HPD (median 1.4 months vs 1.6 months; HR 1.5, 95% CI 1.1–2.2; *p* = 0.02), there was no significant difference in OS between patients with HPD and those with non-HPD (median 5.0 months vs 4.8 months; HR 1.0, 95% CI 0.6–1.5; *p* = 1.0). Furthermore, there was no difference in OS between patients with HPD and those with non-HPD even among the patients with 2 lines of previous chemotherapy (median 7.6 months vs 7.8 months; HR 0.9, 95% CI 0.5–1.6; *p* = 0.6972).

### Clinical outcomes in patients with new lesions in different organs

**Fig. 3:**
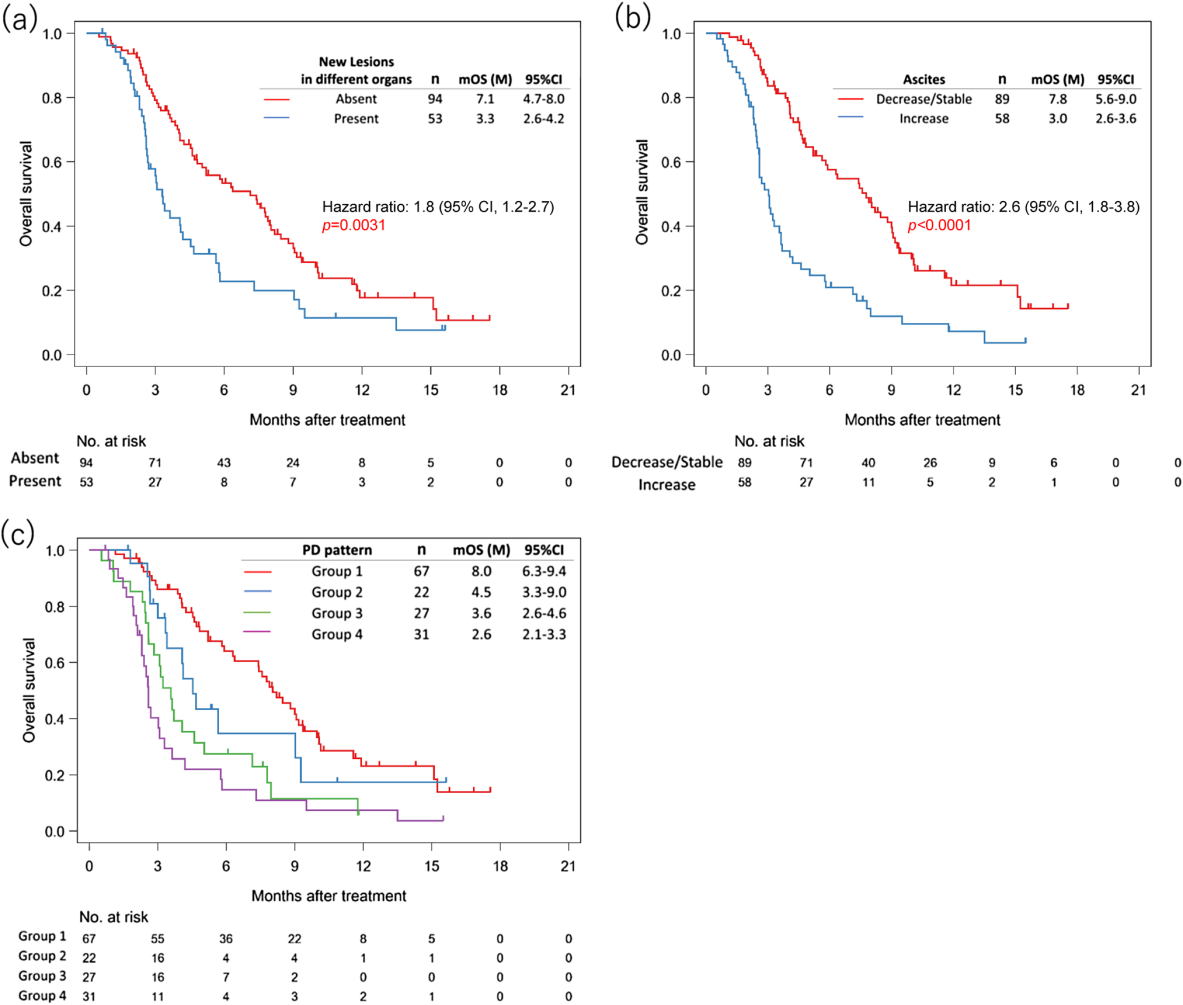
Kaplan–Meier plots showing overall survival (OS). **a** OS curves after initiation of nivolumab according to whether or not new lesions appeared in different organs. Blue lines indicate patients with new lesions in different organs and red lines indicate patients with no such lesions. **b** OS curves after initiation of nivolumab according to whether or not there was a change in amount of ascites. Blue lines indicate patients in whom ascites appeared/increased and red lines indicate patients in whom ascites remained stable/decreased. **c** OS curves after initiation of nivolumab according to whether or not new lesions appeared in different organs and/or ascites appeared/increased: group 1 (−/−), group 2 (+/−), group 3 (−/+), and group 4 (+/+). Red lines indicate patients in group 1, blue lines indicate patients in group 2, green lines indicate patients in group 3, and purple lines indicate patients in group 4

New lesions appeared at the time of disease progression during nivolumab in 73 (49.7%) of 147 patients whose best response was PD. There was no significant difference in median OS between the 73 patients with appearance of new lesions regardless of anatomic sites and the 74 with PD in pre-existing lesions without new lesions (4.1 months vs 6.3 months; HR 1.1, 95% CI 0.8–1.7; *p* = 0.5053) (Supplementary Fig. 1). There was also no significant difference in OS according to the number of new lesions (data not shown). In contrast, new lesions appeared in different organs in 53 of these patients (36.1%) of 147 patients with PD as the best response. OS was significantly worse in the 53 patients in whom new lesions appeared in different organs than in the 94 in whom they did not (median 3.3 months vs 7.1 months; HR 1.8, 95% CI 1.2–2.7; *p* = 0.0031) (Fig. 3a); there were no significant differences in baseline characteristics at initiation of nivolumab between these groups (Supplementary Table 3).

### Clinical outcomes in patients with an appearance/increase of ascites

Ascites appeared/increased in 58 (39.5%) of the 147 patients whose best response was PD; in 29 of these 58 patients, ascites appeared as a new lesion. OS was significantly shorter in these 58 patients with appearance/increase of ascites than in the 89 patients with stable/decreased ascites (median 3.0 months vs 7.8 months; HR 2.6, 95% CI 1.8–3.8; *p* < 0.0001) (Fig. 3b). Large tumor size, presence of peritoneal metastasis, unresectable disease (stage IV), more previous lines of chemotherapy, and high NLR at baseline tended to be more common in the 58 patients with appearance/increase of ascites (Supplementary Table 4). Patients with an increase in ascites had more difficulty receiving subsequent chemotherapy after disease progression than the other groups of patients (51.7% [46/89] vs 75.9% [44/58]; *p* = 0.03). In multivariable analysis, appearance/increase of ascites was associated with inability to receive chemotherapy after disease progression (Supplementary Table 2).

### Clinical outcomes of four patterns of disease progression classified by new lesions in different organs and an appearance/increase of ascites

The 147 patients whose best response was PD were then classified into 4 groups of disease progression patterns according to whether or not new lesions appeared in different organs before initiation of nivolumab and according to whether or not ascites appeared/increased (group 1 [−/−] *n* = 67, group 2 [+/−] *n* = 22, group 3 [−/+] *n* = 27, and group 4 [+/+] *n* = 31). Patients in group 4 tended to have larger tumors, more previous lines of chemotherapy, and a higher NLR at baseline (Table 3). Patients in group 4 had the poorest median OS (2.6 months vs 8.0 months in group 1; HR 3.2, 95% CI 2.0–5.3; *p* < 0.0001) (Table 4, Fig. 3c). Patients were divided into eight groups according to presence or absence of an increase in TGR of ≥ two-fold, new lesions in different organs and appearance/increase of ascites: group 1 [+/+/+], group 2 [+/+/−], group 3 [+/−/+], group 4 [+/−/−], group 5 [−/+/+], group 6 [−/+/−], group 7 [−/−/+] and group 8 [−/−/−] (Supplementary Fig. 2). Comparing between group 1 and 5 (median OS, 2.5 and 2.6 months), between group 2 and 6 (median OS, 4.7 and 4.5 months), between group 3 and 7 (median OS, 3.6 and 3.2 months) and group 4 and 8 (median OS, 8.0 and 8.2 months), there were no differences in OS depending on TGR of ≥ two-fold. The prognosis of AGC patients receiving nivolumab was found to be influenced by new lesions in different organs and appearance/increase of ascites, not by increase in TGR of ≥ two-fold.

**Table 3:** Patient characteristics at initiation of nivolumab according to patterns of progression, whether new lesions appeared in different organs and whether there was appearance/increase of ascites: group (G) 1 (−/−), G 2 (+/−), G 3 (−/+), and G 4 (+/+) among 147 patients whose best response was PD

Total, *n* = 147	G1	G2	G3	G4	*p* value
	*n* = 67	*n* = 22	*n* = 27	*n* = 31	
Sex					
Male	52 (77.6%)	18 (81.8%)	18 (66.7%)	20 (64.5%)	0.22
Female	15 (22.4%)	4 (18.2%)	9 (33.3%)	11 (35.5%)	
Age, years					
Median (range)	69 (41–94)	69 (50–81)	74 (51–84)	67 (29–82)	0.424
Performance status					
0	20 (29.9%)	7 (31.8%)	3 (11.1%)	7 (22.6%)	0.473
≥ 1	45 (67.2%)	15 (68.2%)	24 (88.9%)	24 (77.4%)	
Unknown	2 (2.9%)				
Histological type					
Intestinal	37 (55.2%)	11 (50.0%)	15 (55.6%)	15 (48.4%)	0.518
Diffuse	29 (43.3%)	10 (45.5%)	10 (37.0%)	16 (51.6%)	
Unknown	1 (1.5%)	1 (4.5%)	2 (7.4%)		
HER2 status					
Negative	48 (71.6%)	19 (86.4%)	23 (85.2%)	14 (45.2%)	0.0609
Positive	16 (23.9%)	3 (13.6%)	4 (14.8%)	17 (54.8%)	
Unknown	3 (4.5%)				
Disease status					
Recurrent	28 (41.8%)	12 (54.5%)	8 (29.6%)	7 (22.6%)	0.0739
Stage IV	39 (58.2%)	10 (45.5%)	19 (70.4%)	24 (77.4%)	
Peritoneal metastasis					
No	40 (59.7%)	14 (63.6%)	7 (25.9%)	18 (58.1%)	1
Yes	27 (40.3%)	8 (36.4%)	20 (74.1%)	13 (41.9%)	
Liver metastasis					
No	22 (32.8%)	9 (40.9%)	13 (48.1%)	7 (22.6%)	0.349
Yes	45 (67.2%)	13 (59.1%)	14 (51.9%)	24 (77.4%)	
Metastatic sites, *n*					
1	14 (20.9%)	9 (40.9%)	5 (18.5%)	6 (19.4%)	1
≥ 2	53 (79.1%)	13 (59.1%)	22 (81.5%)	25 (80.6%)	
Previous lines of chemotherapy, n					
< 3	47 (70.1%)	13 (59.1%)	14 (51.9%)	15 (48.4%)	0.045
≥ 3	20 (29.9%)	9 (40.9%)	13 (48.1%)	16 (51.6%)	
Prior ramucirumab treatment					
No	20 (29.9%)	7 (31.8%)	5 (18.5%)	8 (25.8%)	0.597
Yes	47 (70.1%)	15 (68.2%)	22 (81.5%)	23 (74.2%)	
Tumor size, mm					
< 41.3	33 (49.3%)	11 (50.0%)	15 (55.6%)	6 (19.4%)	0.00723
≥ 41.3	34 (50.7%)	11 (50.0%)	12 (44.4%)	25 (80.6%)	
Alkaline phosphatase, U/L					
< 350	39 (58.2%)	14 (63.6%)	14 (51.9%)	15 (48.4%)	0.383
≥ 350	27 (40.3%)	8 (36.4%)	13 (48.1%)	16 (51.6%)	
Unknown	1 (1.5%)				
NLR					
< 1.8	31 (46.3%)	8 (36.4%)	5 (18.5%)	7 (22.6%)	0.0279
≥ 1.8	36 (53.7%)	14 (63.6%)	21 (77.8%)	24 (77.4%)	
Unknown			1 (3.7%)		

*p* value was estimated between G1 and G4

*HER2* human epidermal growth factor receptor 2, *HPD* hyperprogressive disease, *NLR* neutrophil-to-lymphocyte ratio, PD progressive disease

**Table 4:** Comparison of overall survival according to whether new lesions appeared in different organs and/or whether there was appearance/increase of ascites: group (G) 1 (−/−), G 2 (+/−), G 3 (−/+), and G 4 (+/+)

Patients with PD *n* = 147	*n*	Median OS (months)	HR [95% CI]	*p* value
G1	67	8.0 [6.3–9.4]	Reference	
G2	22	4.5 [3.3–9.0]	1.6 [0.9–2.9]	*p* = 0.8461
G3	27	3.6 [2.6–4.6]	2.7 [1.6–4.6]	*p* = 0.0002
G4	31	2.6 [2.1–3.3]	3.2 [2.0–5.3]	*p* < 0.0001

*CI* confidence interval, *HR* hazard ratio, *OS* overall survival, *PD* progressive disease

## Discussion

The main finding in this study was that the pattern of progression associated with poor prognosis in patients with AGC receiving nivolumab whose best response was PD, was appearance of new lesions in different organs and appearance/increase of ascites, not HPD. Appearance/increase of ascites was a particularly important prognostic factor.

In previous studies, HPD during anti-PD-1/PD-L1 therapy has been observed in 9–29% of patients with various types of cancer [20, 22, 23]. The incidence of HPD in patients with AGC has been reported to be 20–30% [24–26] and is associated with poor prognosis. While HPD has also been observed during third-line chemotherapy with irinotecan, the incidence of HPD was found to be higher on nivolumab than on irinotecan and the prognosis was found to be poorer for HPD than for non-HPD in patients on nivolumab but not those on irinotecan [25]. HPD was observed in 16.7% of our patients with AGC treated with nivolumab consistently with previous studies; however, there was no difference in OS between patients with HPD and those with non-HPD. In the ATTRACTION-2 trial which compared nivolumab with placebo as salvage therapy, survival was better from the beginning in the nivolumab group [15] although it is assumed that HPD, if it occurred, might cause more early death in patients receiving nivolumab than in those receiving placebo. Furthermore, a recent report has suggested that disease progression is not more rapid on nivolumab than on placebo [32]. Therefore, it is controversial whether ICIs lead to HPD and poor prognosis in patients with AGC.

Appearance of new lesions was not included in the original definition of HPD, which is based on the sum of the largest diameters of pre-existing measurable lesions. In this study, while new lesions regardless their sites did not have an impact on survival, patients in whom new lesions appeared in different organs had poor prognosis (Supplementary Fig. 1). In contrast, colorectal cancer patients with PD after first-line chemotherapy who developed new lesions regardless the sites were reported to have a significantly poorer prognosis than their counterparts without new lesions [33]. In that study, there was no significant difference in the prognosis between patients with new lesions in different organs and those with new lesions in the pre-existing organs before initiation of chemotherapy. Therefore, debate continues about the clinical significance of the anatomic sites of new lesions. However, in view of previous reports that the number of metastatic sites is a prognostic factor [33] and that progression accompanied by new lesions has a strong negative impact on the prognosis of AGC [27], not including new lesions in the sum of tumor diameters may lead to underestimation of tumor burden. New lesions especially in an organ different from that involved before initiation of nivolumab could be included in the calculation of TGR and HPD in patients with AGC.

Performance status, liver metastases, peritoneal metastases, number of metastatic sites, previous gastrectomy, and the ALP level have been reported to be prognostic factors in patients with AGC on first-line treatment [31, 34, 35]. The peritoneum is one of the most frequent metastatic sites in AGC and becomes increasingly common during the clinical course of the disease. Although peritoneal metastasis is well known to be a poor prognostic factor in AGC, it cannot be measured and is not included in the original definition of HPD. Peritoneal metastasis should be considered when investigating the prognostic impact of the pattern of disease progression in AGC. However, given that it is often difficult to determine progression of peritoneal metastasis objectively, we assessed the appearance/increase of ascites as a surrogate in this study and found that these patients had poor prognosis. Furthermore, in our study, the proportion of patients with appearance/increase of ascites was significantly higher in the group with new lesions in different organs than in the group with new lesions in the same organs (58% [31/53] vs 25% [5/20]; *p* = 0.0183). We suggest that appearance/increase of ascites is a factor that influences the prognosis in patients with AGC. Appearance/increase of ascites may represent progression of peritoneal metastasis and could be taken into consideration as part of HPD in AGC.

Patients with peritoneal metastasis are reported to derive less benefit from nivolumab as third-line therapy than from placebo [36, 37]. The proportion of Treg cells has been found to be higher among lymphocytes in malignant ascites than in peripheral blood [38]. Patients in our group 4, who had both appearance/increase of ascites and new lesions in different organs had the worst prognosis. These patients tended to have larger tumors, more previous lines of chemotherapy, and a higher NLR at baseline. A previous study found that tumor size correlated with low accumulation of tumor-infiltrating lymphocytes and with poor prognosis in patients with AGC [39], while there is a report that clinical failure on blockade of programmed cell death 1 may result from an imbalance between T-cell reinvigoration and tumor burden in melanoma [40]. The NLR is also a predictor response to chemotherapy. PFS and OS have been found to be significantly shorter in patients with AGC who have a high NLR than in those who have a low NLR [41]. Therefore, multiple adverse prognostic factors are likely to affect the outcome of patients with AGC treated with nivolumab.

While it has been reported that subsequent treatment after failure of nivolumab may have a prognostic impact, most of patients in whom there was an increase in ascites could not receive subsequent chemotherapy after disease progression. Many patients whose overall condition deteriorates after disease progression cannot receive treatment regimens such as irinotecan and TFTD (Supplementary Table 2). Because HPD leading to deterioration of patient’s condition and inability to receive subsequent therapy are confounding, it remains unclear how much impacts on poor prognosis the acceleration of tumor growth by itself and worsening of the patient’s condition unfit for subsequent therapy might have respectively.

New aspects of ICIs are becoming apparent as treatment with these agents evolves. In the KEYNOTE-062 trial, Kaplan–Meier estimates showed that survival during the initial 6–9 months was poorer in patients treated with pembrolizumab monotherapy than in those treated with chemotherapy [42]. However, combination of pembrolizumab with chemotherapy reduced the initial mortality [42]. This finding suggests that combination of chemotherapy with an ICI may prevent HPD. Nevertheless, it is important to know the risks and benefits when choosing an ICI for third-line therapy because ICIs have shown efficacy comparable with that of cytotoxic agents in this setting [43]. In present clinical practice, response evaluation with a short interval and careful observation may detect appearance/ increase of ascites not to miss the appropriate timing for switching to subsequent treatment [35] (Table 3, Supplementary Table 3, 4). Given that there are no established biomarkers of an ICI for AGC, combined positive score may select patients who achieve a substantial survival benefit in the first-line treatment. However, there is no biomarkers for negative selection of non-responders, especially for HPD in ICI monotherapy. Therefore, a clinically relevant definition of HPD is essential not only to clarify clinical risk factors and biomarkers predicting HPD but also to develop new treatment to prevent HPD in near future.

This study had several limitations, which stem mainly from its retrospective design. First, we did not compare patients according to whether they received nivolumab or other cytotoxic chemotherapy (irinotecan, TFTD, or T-DXd). Therefore, it is not clear whether the pattern of disease progression associated with poor prognosis found in this study is specific to nivolumab or common with other cytotoxic chemotherapy. Second, numbers of previous chemotherapy differed among the four groups classified by new lesions in different organs and appearance/increase of ascites (Table 3). Prognosis of patients with 3 or more lines of previous chemotherapy was worse than those with 2 lines (Supplementary Table 5) regardless progression pattern. Previous lines of chemotherapy might be a confounding factor of poor prognosis. However, multivariate analysis showed appearance/increase of ascites is an independent factor for not receiving subsequent chemotherapy. Third, the images used to evaluate TGR were obtained by the attending physician as part of routine clinical practice rather than at protocol-specified time points. Therefore, the time interval between successive CT scans differed among the patients. Finally, we did not analyze biomarkers in tissues and blood, which could lead to the discovery of the mechanism(s) of HPD.

In conclusion, the appearance of new lesions in different organs and appearance/increase of ascites, but not the original definition of HPD based on the TGR of pre-existing measurable lesions, were patterns of disease progression associated with poor prognosis in patients with AGC receiving nivolumab monotherapy whose best response was PD. Assuming that mechanism of disease progression accelerated by ICI are common regardless metastatic sites and measurability of lesions, the clinically relevant definition of HPD, including new lesions and non-measurable lesions, should be considered especially for AGC which metastasizes to the peritoneum frequently.

## Supplementary Information

Below is the link to the electronic supplementary material.

Supplementary file1 Supplementary Figure 1. Kaplan-Meier plots showing overall survival (OS). (a) OS curves after initiation of nivolumab according to whether or not new lesions appeared. Blue lines indicate patients with new lesions and red lines indicate patients with no new lesions. (b) OS curves after initiation of nivolumab according to whether new lesions appeared in different organs or in the same organs. Blue lines indicate patients with new lesions in different organs and red lines indicate patients with new lesions in the same organs (DOCX 60 KB)

Supplementary file2 Supplementary Figure 2. Kaplan-Meier plots for overall survival (OS). OS curves after initiation of nivolumab according to presence or absence of an increase in tumor growth rate of ≥ two-fold, new lesions in different organs and appearance/increase of ascites: group 1 [+/+/+], group 2 [+/+/-], group 3 [+/-/+], group 4 [+/-/-], group 5 [-/+/+], group 6 [-/+/-], group 7 [-/-/+] and group 8 [-/-/-]. Red lines indicate patients of group 1, blue lines indicate patients of group 2, green lines indicate patients of group 3, purple lines indicate patients of group 4, orange lines indicate patients of group 5, yellow lines indicate patients of group 6, brown lines indicate patients of group 7 and pink lines indicate patients of group 8 (PDF 375 KB)

## Abbreviations

AGC: Advanced gastric cancer
ALP: Alkaline phosphatase
CPS: Combined positive score
CT: Computed tomography
DCR: Disease control rate
HER2: Human epidermal growth factor receptor 2
HPD: Hyperprogressive disease
HR: Hazard ratio
NLR: Neutrophil-to-lymphocyte ratio
OS: Overall survival
PD: Progressive disease
PFS: Progression-free survival
PR: Partial response
RR: Response rate
SD: Stable disease
T-Dxd: Trastuzumab deruxtecan
TFTD: Trifluridine/tipiracil
TGR: Tumor growth rate
