# Supplementary material for: Pattern of disease progression during third-line or later chemotherapy with nivolumab associated with poor prognosis in advanced gastric cancer: a multicenter retrospective study in Japan
Source: Gastric Cancer. 2022 Nov 1;26(1):132–44. doi: 10.1007/s10120-022-01349-y (PMC9813080; doi:10.1007/s10120-022-01349-y)
Supplement: Supplementary file 1 — Supplementary file1 Supplementary Figure 1. Kaplan-Meier plots showing overall survival (OS). (a) OS curves after initiation of nivolumab according to whether or not new lesions appeared. Blue lines indicate patients with new lesions and red lines indicate patients with no new lesions. (b) OS curves after initiation of nivolumab according to whether new lesions appeared in different organs or in the same organs. Blue lines indicate patients with new lesions in different organs and red lines indicate patients with new lesions in the same organs (DOCX 60 KB) [file 10120_2022_1349_MOESM1_ESM.docx]

Supplementary Table 1. Summary of responses

| Total | n=245 |
| --- | --- |
| CR | 0 (0%) |
| PR | 36 (14.7%) |
| Pseudo progression | 2 (1.0%) |
| SD | 62 (25.3%) |
| PD | 147 (60.0%) |
| HPD | 41 (16.7%) |
| RR (95% CI) | 14.7% (10.5–19.8) |
| DCR (95% CI) | 40.0% (33.8–46.4) |

CR, complete response; DCR, disease control rate; HPD, hyperprogressive disease; PD, progressive disease; PR, partial response; RR, response rate; SD, stable disease

Supplementary Table 2. Multivariate analysis of factors potentially causing inability to receive chemotherapy after disease progression

|  | Received chemotherapy after disease progression | | Multivariate analysis | |  |
| --- | --- | --- | --- | --- | --- |
| n=147 (patients with PD) | No | Yes | OR | 95% CI | p-value |
| HPD status |  |  |  |  |  |
| PD (non-HPD) | 59 (55.7%) | 47 (44.3%) | Reference |  |  |
| HPD | 22 (53.7%) | 19 (46.3%) | 0.908 | 0.42–1.96 | 0.806 |
| Appearance of new lesions in different organs |  |  |  |  |  |
| No | 48 (51.1%) | 46 (48.9%) | Reference |  |  |
| Yes | 33 (62.3%) | 20 (37.7%) | 1.11 | 0.52–2.37 | 0.786 |
| Ascites |  |  |  |  |  |
| Stable/decrease | 38 (42.7%) | 51 (57.3%) | Reference |  |  |
| Appearance/increase | 43 (74.1%) | 15 (25.9%) | 3.74 | 1.76–7.92 | 0.0006 |

CI, confidence interval; HPD, hyperprogressive disease; OR, odds ratio; PD, progressive disease

Supplementary Table 3. Patient characteristics at initiation of nivolumab according to whether or not new lesions appeared in different organs

| New lesions in different organs | Yes | No | p-value |
| --- | --- | --- | --- |
| Patients, n | n=53 | n=94 |  |
| Sex |  |  |  |
| Male | 38 (71.7%) | 70 (74.5%) | 0.703 |
| Female | 15 (28.3%) | 24 (25.5%) |  |
| Age, years |  |  |  |
| Median (range) | 67 (29-82) | 69 (41-94) | 0.283 |
| Performance status |  |  |  |
| 0 | 14 (26.4%) | 23 (24.5%) | 0.846 |
| ≥1 | 39 (73.6%) | 69 (73.4%) |  |
| Unknown |  | 2 (2.1%) |  |
| Histological type |  |  |  |
| Intestinal | 26 (49.1%) | 52 (55.3%) | 0.486 |
| Diffuse | 26 (49.1%) | 39 (41.5%) |  |
| Unknown | 1 (1.9%) | 3 (3.2%) |  |
| HER2 status |  |  |  |
| Negative | 34 (64.2%) | 70 (74.5%) | 0.123 |
| Positive | 19 (35.8%) | 21 (22.3%) |  |
| Unknown |  | 3 (3.2%) |  |
| Disease status |  |  |  |
| Recurrent | 19 (35.8%) | 36 (38.3%) | 0.86 |
| Stage IV | 34 (64.2%) | 58 (61.7%) |  |
| Peritoneal metastasis |  |  |  |
| No | 32 (60.4%) | 47 (50.0%) | 0.234 |
| Yes | 21 (39.6%) | 47 (50.0%) |  |
| Liver metastasis |  |  |  |
| No | 16 (30.2%) | 35 (37.2%) | 0.471 |
| Yes | 37 (69.8%) | 59 (62.8%) |  |
| Metastatic sites, n |  |  |  |
| 1 | 15 (28.3%) | 19 (20.2%) | 0.31 |
| ≥2 | 38 (71.7%) | 75 (79.8%) |  |
| Previous lines of chemotherapy, n |  |  |  |
| <3 | 28 (52.8%) | 61 (64.9%) | 0.164 |
| ≥3 | 25 (47.2%) | 33 (35.1%) |  |
| Prior ramucirumab treatment |  |  |  |
| No | 15 (28.3%) | 18 (19.1%) | 0.221 |
| Yes | 38 (71.7%) | 76 (80.9%) |  |
| Tumor size, mm |  |  |  |
| <41.3 | 19 (35.8%) | 46 (48.9%) | 0.166 |
| ≥41.3 | 34 (64.2%) | 48 (51.1%) |  |
| Alkaline phosphatase, U/L |  |  |  |
| <350L | 30 (56.6%) | 53 (56.4%) | 1 |
| ≥350 | 23 (43.4%) | 40 (42.6%) |  |
| Unknown |  | 1 (1.0%) |  |
| NLR |  |  |  |
| <1.8 | 15 (28.3%) | 36 (38.3%) | 0.281 |
| ≥1.8 | 37 (69.8%) | 58 (61.7%) |  |
| Unknown | 1 (1.9%) |  |  |

HER2, human epidermal growth factor receptor 2; NLR, neutrophil-to-lymphocyte ratio

Supplementary Table 4. Patient characteristics at initiation of nivolumab according to whether ascites appeared/increased or remained stable/decreased

| Ascites | Appearance /increase | Stable/decrease | p-value |
| --- | --- | --- | --- |
| Patients, n | n=58 | n=89 |  |
| Sex |  |  |  |
| Male | 38 (65.5%) | 70 (78.7%) | 0.088 |
| Female | 20 (34.5%) | 19 (21.3%) |  |
| Age, years |  |  |  |
| Median (range) | 73 (29–78) | 69 (41–94) | 0.964 |
| Performance status |  |  |  |
| 0 | 10 (27.5%) | 27 (30.3%) | 0.0802 |
| ≥1 | 48 (72.5%) | 60 (67.4%) |  |
| Unknown |  | 2 (2.3%) |  |
| Histological type |  |  |  |
| Intestinal | 30 (51.7%) | 48 (53.9%) | 0.865 |
| Diffuse | 26 (44.8%) | 39 (43.8%) |  |
| Unknown | 2 (3.4%) | 2 (2.3%) |  |
| HER2 status |  |  |  |
| Negative | 39 (65.8%) | 65 (73.0%) | 0.343 |
| Positive | 19 (34.2%) | 21 (23.6%) |  |
| Unknown |  | 3 (3.4%) |  |
| Disease status |  |  |  |
| Recurrent | 15 (25.9%) | 40 (44.9%) | 0.0236 |
| Stage IV | 43 (74.1%) | 49 (66.1%) |  |
| Peritoneal metastasis |  |  |  |
| No | 25 (43.1%) | 54 (60.7%) | 0.0432 |
| Yes | 33 (56.9%) | 35 (39.3%) |  |
| Liver metastasis |  |  |  |
| No | 20 (34.5%) | 31 (34.8%) | 1 |
| Yes | 38 (65.5%) | 58 (65.2%) |  |
| Metastatic sites, n |  |  |  |
| 1 | 11 (19.0%) | 23 (25.8%) | 0.424 |
| ≥2 | 47 (81.0%) | 66 (74.2%) |  |
| Previous lines of chemotherapy, n |  |  |  |
| <3 | 29 (50.0%) | 60 (67.4%) | 0.0396 |
| ≥3 | 29 (50.0%) | 29 (32.6%) |  |
| Prior ramucirumab treatment |  |  |  |
| No | 13 (22.4%) | 20 (22.5%) | 1 |
| Yes | 45 (77.6%) | 69 (77.5%) |  |
| Tumor size, mm |  |  |  |
| <41.3 | 19 (32.8%) | 46 (51.7%) | 0.0279 |
| ≥41.3 | 39 (67.2%) | 43 (48.3%) |  |
| Alkaline phosphatase, U/L |  |  |  |
| <350 | 29 (50.0%) | 54 (60.7%) | 0.232 |
| ≥350 | 29 (50.0%) | 34 (38.2%) |  |
| Unknown |  | 1 (1.1%) |  |
| NLR |  |  |  |
| <1.8 | 12 (20.7%) | 39 (3.8%) | 0.00436 |
| ≥1.8 | 46 (79.3%) | 49 (55.1%) |  |
| Unknown |  | 1 (1.1%) |  |

HER2, human epidermal growth factor receptor 2; NLR, neutrophil-to-lymphocyte ratio

Supplementary Table 5. Prognostic impact of the number of previous chemotherapy according to the progression patterns

| Progression pattern | Number of previous chemotherapy | n | Median OS (months) (95%CI) | HR (95%CI) | p-value |
| --- | --- | --- | --- | --- | --- |
| All patients | 2 | 152 | 9.1 (8.0-11.6) | reference |  |
|  | ≥3 | 93 | 5.7 (4.1-9.3) | 1.5 (1.1-2.0) | 0.02368 |
| HPD | 2 | 25 | 7.6 (4.5-10.1) | reference |  |
|  | ≥3 | 16 | 3.1 (1.8-5.0) | 3.0 (1.3-6.5) | 0.007501 |
| Non-HPD | 2 | 64 | 7.8 (4.8-8.8) | reference |  |
|  | ≥3 | 42 | 3.7 (2.7-4.5) | 1.8 (1.1-2.7) | 0.01411 |
| New lesions in different organs | 2 | 28 | 5.8 (2.7-9.3) | reference |  |
|  | ≥3 | 25 | 2.7 (2.3-3.3) | 2.5 (1.3-4.7) | 0.006877 |
| No new lesions  in different organs | 2 | 61 | 7.9 (5.8-9.0) | reference |  |
|  | ≥3 | 33 | 4.1 (3.1-6.4) | 1.6 (1.0-2.6) | 0.07055 |
| Appearance/Increase of ascites | 2 | 29 | 3.6 (2.4-7.3) | reference |  |
|  | ≥3 | 29 | 2.8 (2.4-3.2) | 1.8 (1.0-3.2) | 0.04493 |
| No appearance/increase of ascites | 2 | 60 | 8.5 (7.4-9.3) | reference |  |
|  | ≥3 | 29 | 4.5 (3.4-6.4) | 1.7 (1.0-2.9) | 0.05953 |

CI, confidence interval; HPD, hyperprogressive disease; HR, hazard ratio; OS, overall survival
