# Supplementary figures and images for: Pattern of disease progression during third-line or later chemotherapy with nivolumab associated with poor prognosis in advanced gastric cancer: a multicenter retrospective study in Japan
Source: Gastric Cancer. 2022 Nov 1;26(1):132–44. doi: 10.1007/s10120-022-01349-y (PMC9813080; doi:10.1007/s10120-022-01349-y)

(a)

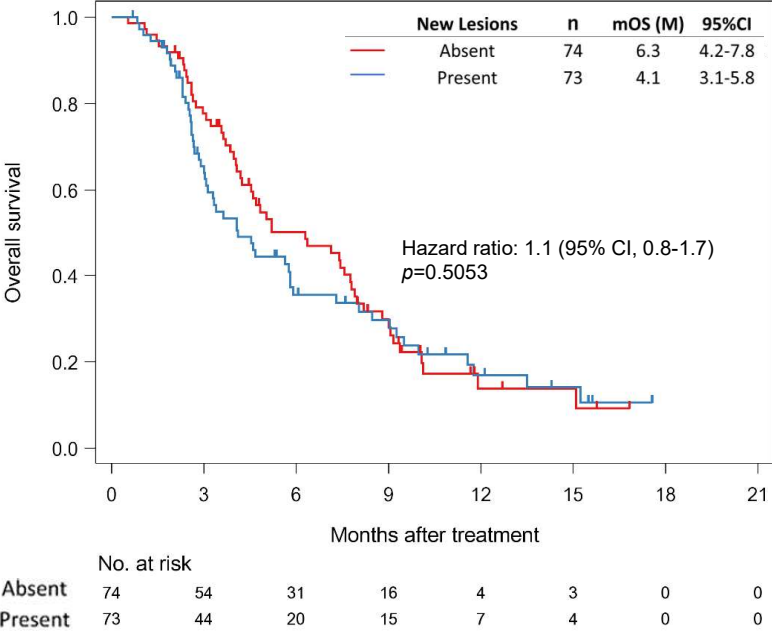

(b)

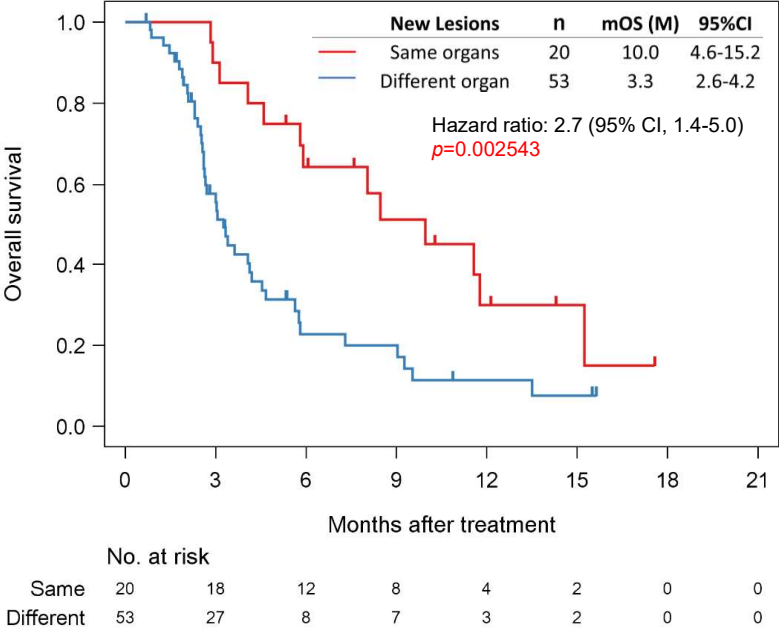

Supplementary Figure 1a.1b.

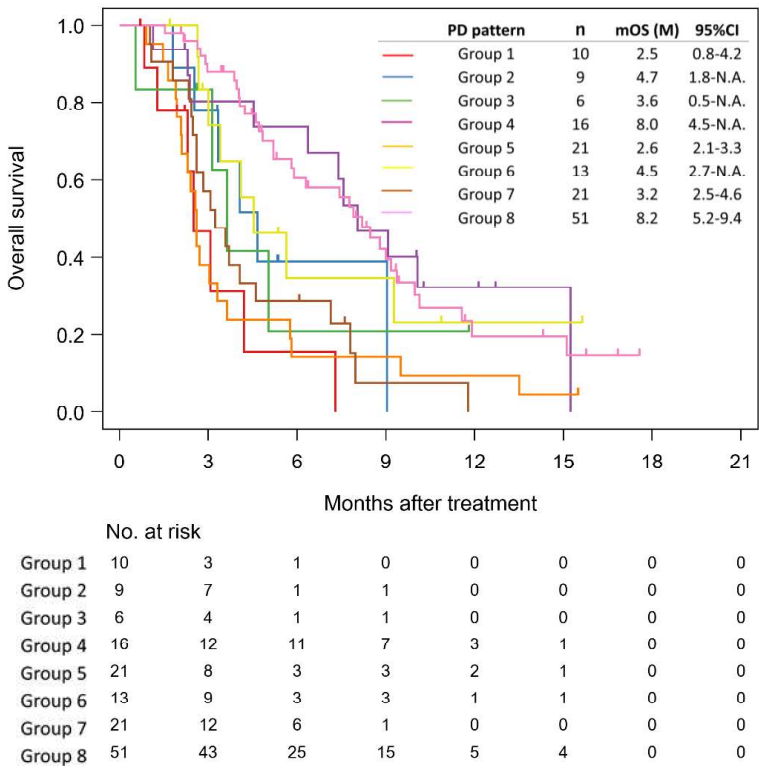

Supplementary Figure 2.

Supplement: Supplementary file 2 — Supplementary file2 Supplementary Figure 2. Kaplan-Meier plots for overall survival (OS). OS curves after initiation of nivolumab according to presence or absence of an increase in tumor growth rate of ≥ two-fold, new lesions in different organs and appearance/increase of ascites: group 1 [+/+/+], group 2 [+/+/-], group 3 [+/-/+], group 4 [+/-/-], group 5 [-/+/+], group 6 [-/+/-], group 7 [-/-/+] and group 8 [-/-/-]. Red lines indicate patients of group 1, blue lines indicate patients of group 2, green lines indicate patients of group 3, purple lines indicate patients of group 4, orange lines indicate patients of group 5, yellow lines indicate patients of group 6, brown lines indicate patients of group 7 and pink lines indicate patients of group 8 (PDF 375 KB) [file 10120_2022_1349_MOESM2_ESM.pdf]
